# Supplementary material for: Age Associated Decrease of MT-1 Melatonin Receptor in Human Dermal Skin Fibroblasts Impairs Protection Against UV-Induced DNA Damage
Source: Int J Mol Sci. 2020 Jan 3;21(1):326. doi: 10.3390/ijms21010326 (PMC6982064; doi:10.3390/ijms21010326)
Supplement: Supplementary file 1 [file ijms-21-00326-s001.pdf]

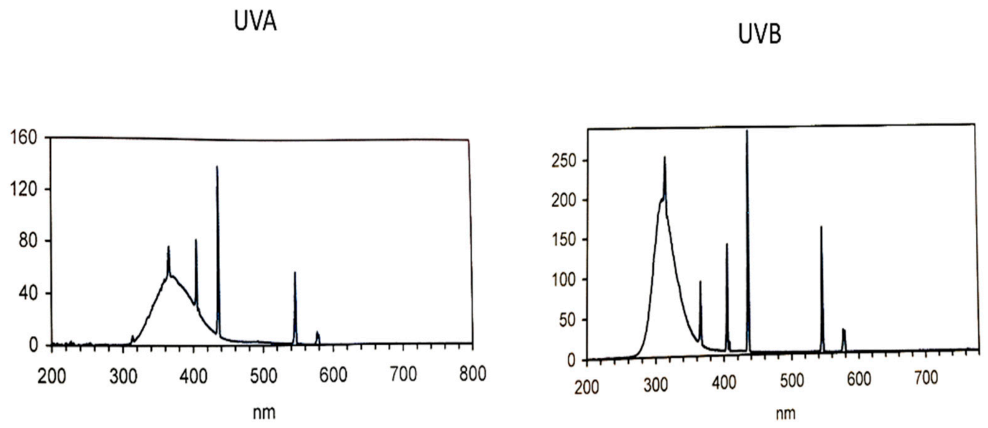

Supplementary Figure 1: UVR spectra from the Dr. Gröbel irradiation chamber fitted with UVA (F15T8BLB) and UVB (G15T8E) bulbs.
